# *Plasmodium falciparum* gametocyte-induced volatiles enhance attraction of *Anopheles* mosquitoes in the field

**DOI:** 10.1186/s12936-020-03378-3

**Published:** 2020-09-04

**Authors:** Yared Debebe, Sharon Rose Hill, Göran Birgersson, Habte Tekie, Rickard Ignell

**Affiliations:** 1 Department of Zoological Sciences, Addis Ababa University, PO. Box 1176, Addis Ababa, Ethiopia; 2 Unit of Chemical Ecology, Department of Plant Protection Biology, Swedish University of Agricultural Sciences, PO. Box 102, Alnarp, Sweden

**Keywords:** (*E*)-4-hydroxy-3-methyl-but-2-enyl pyrophosphate, HMBPP, *Anopheles*, Mosquitoes, Attraction

## Abstract

**Background:**

*Plasmodium* parasites manipulate the interaction between their mosquito and human hosts. Patients infected with gametocytes attract anopheline mosquitoes differentially compared to healthy individuals, an effect associated with an increased release of attractive volatile cues. This odour-driven manipulation is partly mediated by the gametocyte-specific metabolite, (*E*)-4-hydroxy-3-methyl-but-2-enyl pyrophosphate (HMBPP), which induces increased release of select aldehydes and terpenes from red blood cells and results in the enhanced attraction of host-seeking mosquitoes, which are vectors of malaria. This study investigates the effect of the HMBPP-induced volatiles on the attraction of wild *Anopheles* mosquitoes to humans under field conditions.

**Methods:**

The efficacy of the HMBPP-induced odour blend to attract *Anopheles* was evaluated in a 4 × 6 Latin rectangular study design indoors using baited Suna traps. Furthermore, to assess the efficacy of the HMBPP-induced odour blend in (1) augmenting the attractiveness of human odour, and (2) attracting *Anopheles* mosquitoes in the absence of human odour, a two-choice assay using host decoy traps (HDTs) was used and evaluated using binomial generalized regression.

**Results:**

Traps baited with the HMBPP-induced odour blend attracted and caught both *Anopheles arabiensis* and *Anopheles pharoensis* females in a dose-dependent manner in the presence of background human odour, up to 2.5 times that of an unbaited trap. Given a choice between human odour and human odour laden with the HMBPP-induced odour blend, mosquitoes differentially preferred traps augmented with the HMBPP-induced odour blend, which caught twice as many female *An. arabiensis*. Traps baited with the HMBPP-induced odour blend but lacking the background of human odour were not effective in attracting and catching mosquitoes.

**Conclusion:**

The findings of the present study revealed that the HMBPP-induced odour blend, when augmented with human body odour, is attractive to anopheline mosquitoes and could be used as a complementary vector control tool along with existing strategies.

## Background

Malaria parasites manipulate both their mosquito and human hosts to increase the interactions between them, thereby enhancing the risk of transmission [1–6]. The presence of *Plasmodium falciparum* and *Plasmodium vivax* gametocytes, the transmissible stage of the parasite, manipulates the host-seeking behaviour of its primary vectors in sub-Saharan Africa, *Anopheles gambiae *sensu lato [1, 2, 4–6] and in South America, *Anopheles darlingi* [3]. This manipulation is most clearly observed in the change in host odour profile of gametocyte-infected patients, resulting in the doubling of the attractiveness of these patients to malaria vectors, compared to uninfected healthy persons [1, 2, 4–7]. A plausible mechanism underlying this altered attraction was described recently [5–8]. However, as of yet, there has been no evaluation of malaria parasite-induced volatiles in the field and how these affect the attractiveness of humans to mosquitoes, which vector malaria.

Emami et al*.* [8] recently reported that the *P. falciparum* metabolite, (*E*)-4-hydroxy-3-methyl-but-2-enyl pyrophosphate (HMBPP), both directly and indirectly manipulated vector behavior. HMBPP induced an increased feeding rate and indirectly stimulated attraction of *An. gambiae *sensu stricto to red blood cells, by enhancing the release of select aldehydes and monoterpenes. When combined in a blend, these volatile compounds reproduced the behavioral response of *An. gambiae* to gametocyte-infected red blood cells*,* when they were presented in the background of red blood cell odour [8]. These volatiles were hypothesized to be emitted from gametocyte-infected persons [8], and subsequent studies have recently demonstrated an altered profile of these, and other, volatiles emanating from both the breath and skin of gametocyte-infected children [4–7].

In this study, the efficacy of the HMBPP-induced odour blend in attracting wild *Anopheles* mosquitoes was evaluated. The results obtained demonstrated that host-seeking anopheline mosquitoes have a higher propensity to select human body odour augmented with the HMBPP-induced blend under field conditions. The perspective of using the findings of this study towards surveillance and integrated vector management are discussed.

## Methods

### Study site description

**Fig. 1:**
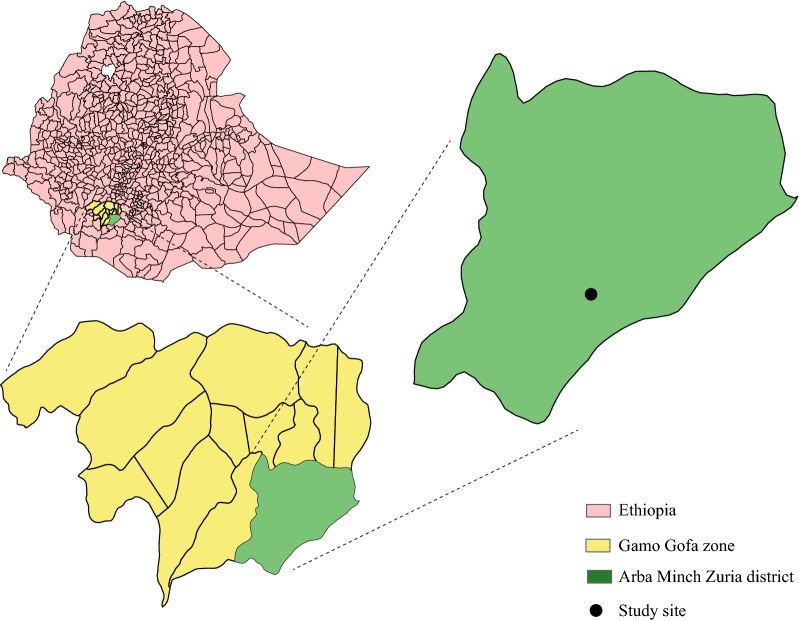
Map of the study area showing the districts in Ethiopia, the Gamo Gofa zone and the Arba Minch Zuria district, with the study site indicated

The study was conducted in Arba Minch Zuria district of the Gamo Gofa zone in southern Ethiopia, at the outskirts of a village called Sile (5°53′24″ N, 37°29′24″ E; Fig. 1). Detailed description of the study area is outlined by Debebe et al*.* [9]. The area is predominantly covered by banana plantations, which are a major source of income for the residents. Maize and cotton are also cultivated. The houses in this area are transiently inhabited by farmers during the seasons for cultivation, during which time the occupancy in each house is one to two persons, predominantly men between the ages of 18–30. The area is one of the most malarious in the district, with *An. arabiensis* as the dominant vector and *An. pharoensis* playing a secondary role in malaria transmission [9, 10].

### Attraction of HMBPP-induced volatiles

To assess the attractiveness of the HMBPP-induced odour blend, a 4 × 6 Latin rectangle study design was used. For the study, 12 traditionally built houses with mud walls and grass thatched roofs were selected. Houses were separated by a minimum of 200 m. The 12 houses were divided into three groups in which the three treatments and a control were randomly assigned on the first night. Treatments and controls were rotated nightly, so that each visited each house six times over the course of 24 nights. Three serial dilutions of the odour blend (treatments) and heptane (control; Merck, Darmstadt, DE) were dispensed using wick dispensers, allowing for the release of all of the compounds in constant ratios throughout the 12 h assay [11]. The experiment was conducted using Suna traps (BioGents AG, Regensburg, DE) hung 20 cm above the ground near the foot of a sleeping, asymptomatic person under a bed net, i.e. both treatments and control were tested against the background of human odour. While the volunteers were not tested for malaria prior to the study, the presence of an asymptomatic malaria-infected person cannot be excluded, and thus increases the probability of a type II error. The traps were set to run from 6:00 p.m. to 6:00 a.m., the main activity period of the *Anopheles* mosquitoes in the area. The study was conducted during the long rainy season in July–August 2019. The odour blend mimicked the composition and ratio of compounds (( ±)-α-pinene:(−)-β-pinene:(±)-limonene:octanal:nonanal:decanal; 1:0.7:2.5:3.1:48:1.6) emanating from gametocyte-infected red blood cells and induced by the metabolite HMBPP [8]. The odour blend was serially diluted in heptane at 1.0, 10, and 100 ng µl^−1^ resulting in release rates of 1.6, 16 and 164 ng min^−1^ of ( ±)-α-pinene, respectively. Based on the average release rate of the majority of these compounds from healthy volunteers [12], these release rates correspond to a ca. 1, 10 and 100 times increase over that of the sleeping volunteer. In comparison, Emami et al*.* [8] reported a relative increase of the HMBPP-induced volatiles of 1.2–5.2 from 1 ml of red blood cells treated with 10 µM HMBPP.

### Differential attraction to HMBPP-induced volatiles

To evaluate the effect of the HMBPP-induced volatiles on the relative attractiveness of two asymptomatic individuals, with rapid diagnostic test (RDT) negative peripheral blood status (CareStart™ Pf/Pv (HRP2/pLDH) Ag Combo RDT, [13]), a two-choice assay was used. It is important to note that this RDT is 88.8% and 77.6% accurate for identifying *P. falciparum* and *P. vivax* infections, respectively [14], and as such, asymptomatic cases of malaria may have been overlooked. Two experiments were conducted: (1) two tents, each with a human volunteer as a source of human odour; and (2) two tents without human volunteers. In both cases, the tents (Fig. 3a, b) were separated by at least 200 m. Two polyvinylchloride pipes (10 m length × 10 cm diameter) extending from each tent carried the odour from the tents, with the help of battery-operated fans (12 V) at the mouth of the pipe within the tent (Fig. 3a, b). The odour blend was delivered to host decoy traps (HDTs; BioGents AG; [15]), which were filled with heated water and covered in adhesive-coated transparent plastic sheeting, as per manufacturer’s instructions, and placed 10 cm from the exhaust (Fig. 3a). During the first experimental night, the volunteers were randomly assigned to sleep in either tent and then the wick dispensers containing either the odour blend with the highest release rate, identified above, or heptane, were placed 10 cm inside the mouth of each plastic pipe, downwind of the fan (Fig. 3a, b). A similar protocol was used in the control experiments, without the sleeping volunteers. For all four tents, the control and the odour blend were rotated between the pipes every night to account for positional bias of the treatments. In the two inhabited tents, the volunteers were rotated between the tents every other night after one full round of the experiment, to account for differences in the odour profiles of the volunteers. Both experiments were conducted for a total of eight nights.

### Sorting and identification of mosquitoes

Captured *Anopheles* mosquitoes were transported to the field laboratory and sorted according to physiological state, as unfed, engorged, semi-gravid and gravid, following the categories outlined by WHO [16]. Morphological species identification of the adult female *Anopheles* mosquitoes was conducted using identification keys developed by Verrone [17]. Mosquitoes belonging to the *An. gambiae* species complex were considered as *An. arabiensis* since a recent study has confirmed that *An. arabiensis* is the only member of the *An. gambiae* species complex in the area [9].

### Data analysis

The effect of the different release rates of the odour blend and the control on the daily numbers of *An. arabiensis* and *An. pharoensis* caught in the traps was estimated using zero-inflated negative binomial regression of the generalized linear model (GLM; JMP Pro version 13 SAS Institute Inc., Cary, NC, USA). The control group was used as a reference category in both analyses and a Dunnett’s *post-hoc* test was used to compare each treatment group with the control.

The relative attractiveness of the odour blend with respect to the control was analysed using a negative binomial regression of the GLM (JMP Pro v. 13), taking into account the volunteers and treatments as factors. To visualize the results, choice indices were calculated by dividing the number of anophelines caught in either the HMBPP-induced odour baited traps (T) or the control trap (C) by the total number of mosquitoes caught in both traps (T + C). Pairwise comparisons were made using Tukey’s post hoc test. All tests were computed at the significance level of α < 0.05.

## Results

### HMBPP-induced volatiles attract *Anopheles* mosquitoes

A total of 2114 *Anopheles* mosquitoes were captured in odour-baited and control traps, belonging to two species, *An. arabiensis* and *An. pharoensis*, over the 24 experimental nights. *Anopheles arabiensis* was the predominant species, with a total of 1931 (91.3%) individuals caught, with the remaining mosquitoes identified as *An. pharoensis* (183 individuals; 8.7%). Approximately 93.6% and 95% of *An. arabiensis* and *An. pharoensis*, respectively, were host-seeking, while the remaining mosquitoes were blood fed.

**Fig. 2:**
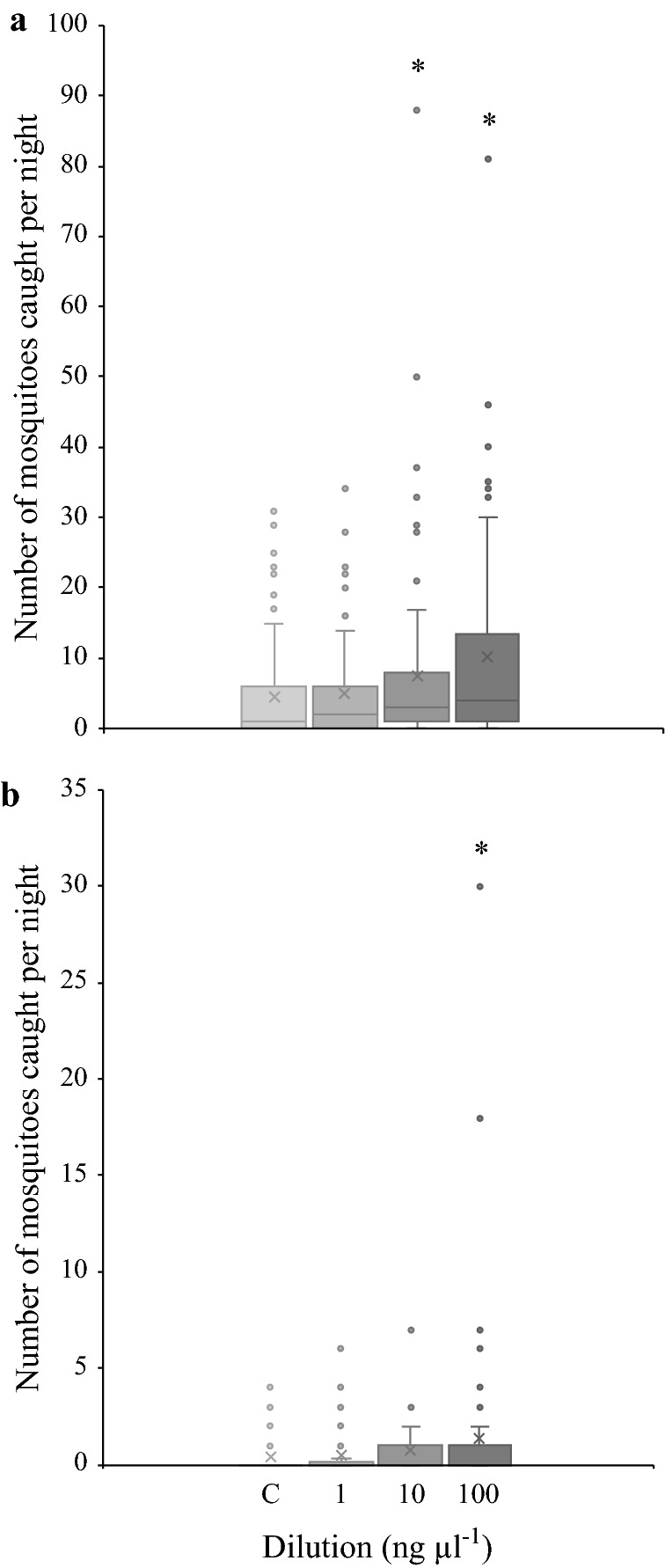
The number of *Anopheles arabiensis* (**a**) and *Anopheles pharoensis* (**b**) caught in the HMBPP-induced odour-baited and control traps per night as depicted by box plots. The horizontal line within each box represents the median and the × represents the mean. The whiskers represent ± 1.5 times the inter-quartile range extending from the upper and lower quartile boundaries. The asterisks represent significant difference from the control at a level of α < 0.05 (N = 24; n = 72)

While the number of *An. arabiensis* captured per night significantly increased with an increasing release rate of the HMBPP-induced odour blend (GLM; χ^2^ = 12.89; P = 0.0049), there was no overall effect of release rate on the number of *An. pharoensis* caught (GLM; χ^2^ = 6.84; P = 0.077; Fig. 2). A post hoc analysis revealed that the number of *An. arabiensis* captured in the traps baited with the two highest release rates of the HMBPP-induced odour blend was significantly higher than that of the control (Fig. 2a). In addition, the post hoc analysis revealed that a significantly higher number of *An. pharoensis* were caught in traps with the highest release rate of the blend (Fig. 2b).

### HMBPP-induced volatiles differentially attract *Anopheles arabiensis*

**Fig. 3:**
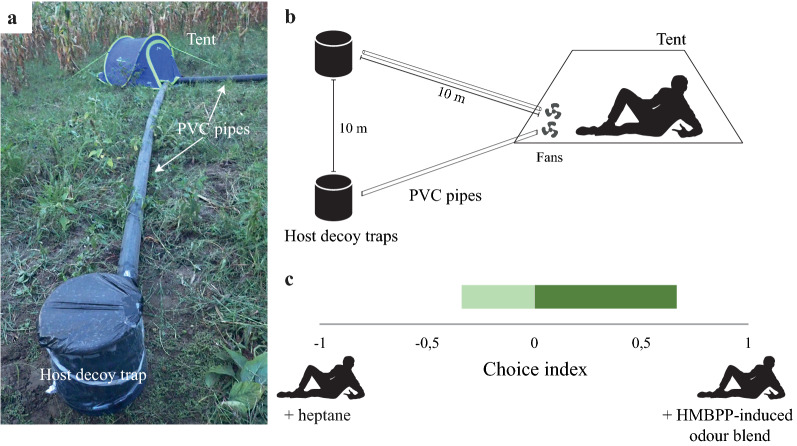
**a** Photograph of the tent and one of the two BioGents AG host decoy traps connected by a polyvinylchloride (PVC) pipe. **b** Schematic representation of the two-choice behavioral assay, indicating the volunteer inside the tent. The human body odours were deployed to the host decoy traps via PVC pipes using two computer fans. The HMBPP-induced odour blend and the heptane control were introduced into either of the two PVC pipes by placing the dispensers in front of the fans. **c** The behavioral preference of *Anopheles arabiensis* to human odour, from two volunteers with or without augmentation by the HMBPP-induced odour blend is indicated by a choice index. In the absence of human odour, the host decoy traps caught 3 mosquitoes in the treatment and 4 in the control (data not shown). All experiments were replicated 8 times

A total of 166 *An. arabiensis* were caught by the human decoy traps over the eight experimental nights. The HMBPP-induced odour blend caught significantly more *An. arabiensis* in the presence of human odour (χ^2^ = 5.63; df = 1; P < 0.018), with almost twice as many mosquitoes caught in the HDT baited with HMBPP-induced volatiles (total: 113; median: 6 mosquitoes per night; IQR = 7.5; mean 7.1 ± 1.35 mosquitoes per night) compared to the heptane control (total: 53; median: 2 mosquitoes per night; IQR = 5.00; mean: 3.3 ± 0.76 mosquitoes per night; Fig. 3c). There was no significant difference found between the number of mosquitoes caught for either volunteer (χ^2^ = 0.026; df = 1; P = 0.8). In the absence of human odour, the HDT captures were very low, with 4 mosquitoes caught in the control and 3 in the treatment, with no significant difference between the treatment and control traps (χ^2^ = 0.11; df = 1; P = 0.73).

## Discussion

The aldehydes and monoterpenes induced by *P. falciparum* gametocytes and the metabolite HMBPP, and released by red blood cells, attracts wild female *Anopheles* mosquitoes and augments the attractiveness of healthy human volunteers under field conditions. The results of this study demonstrate that a blend of volatile compounds reflecting the odours induced by *P. falciparum* gametocyte parasites in the red blood cells, and emitted by an infected person, are able to manipulate the host-seeking behaviour of malaria vectors in the background of human odour, thereby increasing vectorial capacity. While interesting from a fundamental perspective, these results have implications for monitoring in the context of integrated vector management.

In this study, traps baited with the HMBPP-induced odour blend differentially augmented the attractiveness of humans to *An. arabiensis* by up to two times, reflecting a similar increased behavioral response to patients with gametocyte infection for both *P. falciparum* and *P. vivax* [1, 3, 4]. As HMBPP is a gametocyte-specific *Plasmodium* metabolite [8], and the increased attraction of mosquitoes corresponds with the gametocyte stage of infection [1, 3, 4], it is likely that the HMBPP production by gametocytes underpins the observed increase in vector attraction to subjects with gametocyte infection. The volatiles that increase 1.2 to 5.2 times in emission from red blood cells, induced by either gametocyte infection or the presence of HMBPP, include the monoterpenes, α-pinene, ß-pinene and limonene, as well as the aldehydes, octanal, nonanal and decanal [8]. The release rates of the HMBPP-induced odour blend assessed in this study were chosen to enhance the natural emission of these compounds in human body odour [12] by approximately 1, 10 and 100 times to reflect those from *P. falciparum* gametocyte-infected red blood cells. This largely corresponds with those volatiles observed to increase in emission from the skin and in the breath of *P. falciparum*-infected children, compared to children post-malaria treatments, with discrepancies possibly due to differences in volatile collection methodology [6, 7]. Interestingly, in the absence of the human odour, the HDT failed to attract mosquitoes, indicating that heat and high contrast, by themselves, are not sufficient to drive attraction. This effect could not be rescued by the addition of the HMBPP-induced odour blend, which also did not elicit attraction of *Anopheles* in the absence of the human odour, emphasizing the requirement of human background odour to induce attraction, and thus, effective captures with these baited traps.

## Conclusions

The present study revealed that the HMBPP-induced odour blend, when augmented with human body odour, attracts wild female *Anopheles* mosquitoes under field conditions. However, a more extensive analysis, including a broader set of volunteers with defined malaria status at multiple locations, is required to assess the robustness of this finding. The requirement of the human background odour for attraction, although constraining the use of the HMBPP-induced blend, opens novel ways to survey and control mosquitoes, which vector malaria, both indoors and outdoors. While it is plausible to direct human laden air from houses, supplemented with the HMBPP-induced blend, to HDT-type outdoor traps [15], the combination of the HMBPP-induced blend with eave tube-type control technology (e.g. [18]) might be more cost effective and more manageable at the household and community levels. Alternatively, the HMBPP-induced blend could be used in conjunction with synthetic human blends, e.g. the MB5 blend, which has been shown to be highly effective in attracting and controlling mosquitoes, which transmit malaria, outdoors [19]. A remaining challenge in this regard would be the development of long-lasting formulations of the odour blend.

## Data Availability

All data generated or analysed during this study are included in this published article.

## Abbreviations

HDT: Host decoy trap
HMBPP: (*E*)-4-hydroxy-3-methyl-but-2-enyl pyrophosphate
